# The Antioxidant, Anti-Inflammatory and Immunomodulatory Effects of Camel Milk

**DOI:** 10.3389/fimmu.2022.855342

**Published:** 2022-04-12

**Authors:** Sepide Behrouz, Saeideh Saadat, Arghavan Memarzia, Hadi Sarir, Gert Folkerts, Mohammad Hossein Boskabady

**Affiliations:** ^1^Department of Animal Science, Faculty of Agriculture, University of Birjand, Birjand, Iran; ^2^Department of Physiology, School of Medicine, Zahedan University of Medical Sciences, Zahedan, Iran; ^3^Applied Biomedical Research Center, Mashhad University of Medical Sciences, Mashhad, Iran; ^4^Department of Physiology, Faculty of Medicine, Mashhad University of Medical Sciences, Mashhad, Iran; ^5^Division of Pharmacology, Utrecht Institute for Pharmaceutical Sciences (UIPS), Faculty of Science, Utrecht University, Utrecht, Netherlands

**Keywords:** anti-inflammatory, antioxidant, camel milk, immunomodulatory, treatment

## Abstract

Camel milk (CM) has been found to have several health benefits, including antiviral, antibacterial, anti-tumor, anti-fungal, antioxidant, hypoglycaemic and anti-cancer activities. In addition, CM can counter signs of aging and may be a useful naturopathic treatment for autoimmune diseases. The composition of CM varies with geographic origin, feeding conditions, seasonal and physiological changes, genetics and camel health status. In the present review, we collate the diverse scientific literature studying antioxidant, anti-inflammatory and immunomodulatory effects of CM and its bioactive compounds. The databases Scopus, PubMed, and Web of Science were searched until the end of September 2021 using the keywords: camel milk, antioxidant, anti-inflammatory, immunomodulatory. The anti-inflammatory mechanism of CM in various inflammatory disorders was consistently reported to be through modulating inflammatory cells and mediators. The common anti-inflammatory bioactive components of CM seem to be lactoferrin. The antioxidant effects of α-lactalbumin, β-caseins and vitamin C of CM work by reducing or inhibiting the production of reactive oxygen species (ROS), hydroxyl radicals, nitric oxide (NO), superoxide anions and peroxyl radicals, likely alleviating oxidative stress. Higher levels of protective proteins such as lysozyme, IgG and secretory IgA compared to cow’s milk, and insulin-like protein activity of CM on ß cells appear to be responsible for the immunomodulatory properties of CM. The evidence indicates that CM and its bioactive components has the potential to be a therapeutic value for diseases that are caused by inflammation, oxidative stress and/or immune-dysregulation.

## Introduction

Camels are capable of producing 4 to 30 litres of milk per day in unfavourable conditions such as extreme temperatures, lack of pasture, and lack of water (1). Their lactation period is between 9 to 11 months with a maximum lactation of 2 to 3 months. Hence, in many arid and semi-arid regions of the world, camels are a primary source of milk and meat (1). In fact, camel milk (CM) is one of the most essential components of the human diet in these areas (1).

Through their consumption of CM, people in arid regions are aware of the health benefits (2), as well as the nutritional benefits of CM, and often refer to CM as white desert gold. CM is used to treat various infections, jaundice, asthma and high blood pressure (3), and is also known to positively regulate blood sugar levels (4), including in patients of diabetes (5).

Moreover, CM is known to have antiviral, antibacterial, anti-tumor, anti-fungal, antioxidant, hypoglycemic and anti-cancer activities, prevents the effects of aging on health and reduces the symptoms of autoimmune diseases (6, 7). CM also affects numerous biological activities such as metabolic responses to absorb nutrients, digestion, growth and development of specific organs and disease resistance (8). Protective proteins in CM are key to boosting the body’s immune defense mechanisms, particularly antibacterial and antiviral activity (9). For example, antimicrobial activity of CM is related to high levels of protective proteins such as lactoferrin (Lf), immunoglobulin (Ig) IgGs, lactoperoxidase, lysozyme, peptidoglycan recognition protein-1 (PGRP-1) and other enzymes (9, 10).

Although there is a significant body of research into the potential health benefits of CM, an in-depth review into the biological mechanisms of CM therapeutic effects has not been published. Here, we describe in detail the composition of CM and then discuss the research findings into the anti-inflammatory, antioxidant and immunomodulatory effects of CM.

## Methods

The databases Scopus, PubMed and Web of Science were searched using the keywords: camel milk, antioxidant, anti-inflammatory and immunomodulatory, including studies up to the end of September 2021.

For inclusion of papers in the current review, the eligibility criteria were (1): *in vitro*, animal and clinical trials that investigated (2) the supplementation of CM and its dosage (3), treatment of CM alone or in combination with other compounds and (4) the antioxidant, anti-inflammatory and immunomodulatory effects of CM.

The exclusion criteria in this review were (1): the absence of CM supplementation alone or in combination with other compounds or studies that investigated (2) other health benefits of CM outside of its antioxidant, anti-inflammatory and immunomodulatory properties. Articles in a language other than English were also excluded.

**Figure 1:**
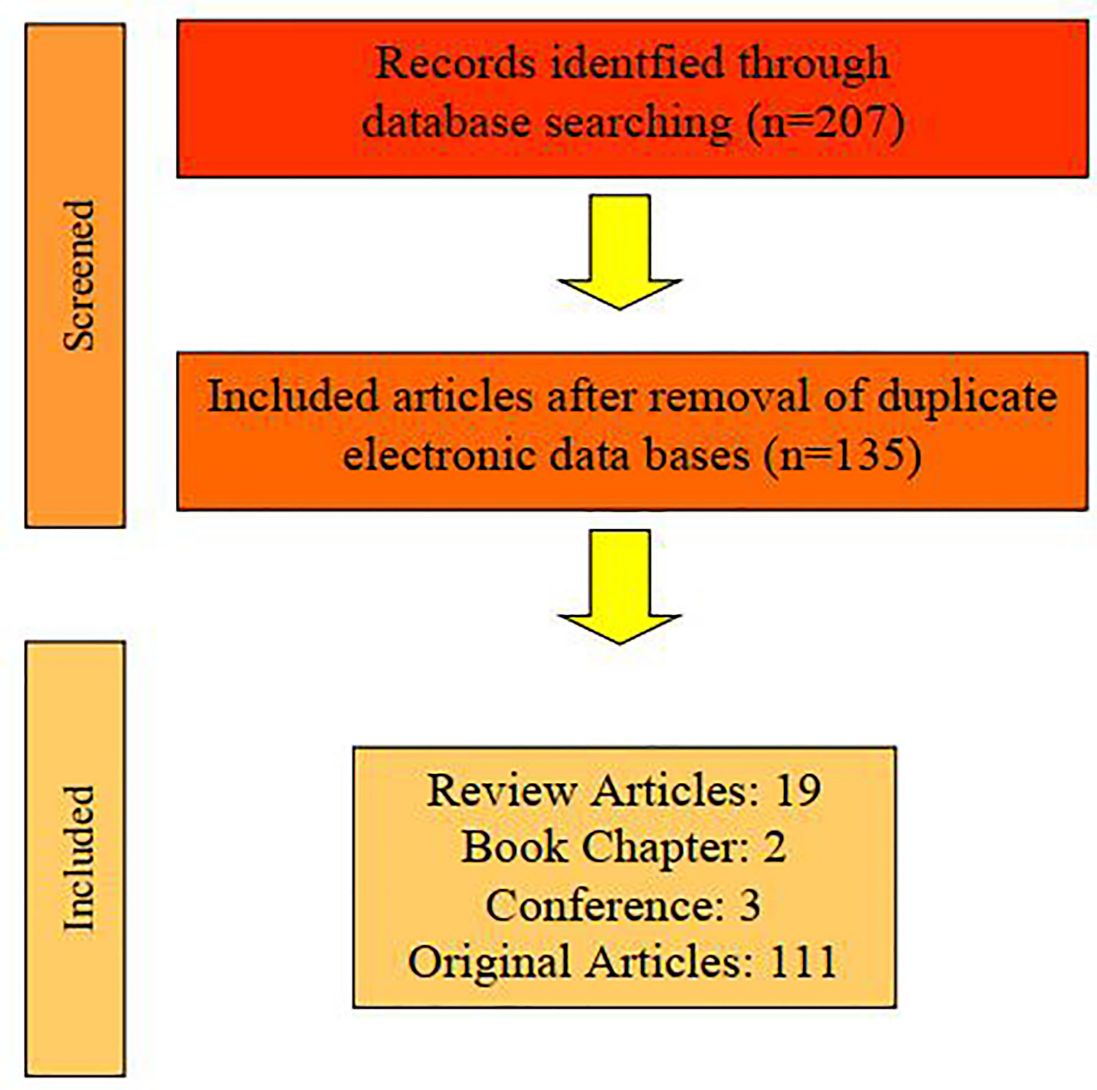
Flowchart of searching and selecting articles for the current review.

In total, 175 articles were selected and 96 (16 reviews, 2 book chapter and 78 original articles) were included in this review because 79 articles were duplicates. The article searching flowchart is shown in **Figure 1**.

## The Composition Of CM

CM tends to have a matte white color, is sweet in taste, although sometimes salty, and has a sharp smell (11). The salty taste of CM is due to the high levels of chloride and the significant amount of iron, copper and phosphorus (12). The composition of CM varies between geographic locations and other factors such as stage of postpartum, feeding conditions, seasonal and physiological changes, individual genetics and health status. Nonetheless, the macro-composition of CM is 86.3-88.5% water, 2.9-5.5% fat, 2.5-4.5% protein, 8.9-14.3% milk solids, 0.35-0.95% ash and 2.9-5.8% lactose (13). The composition of milk in different species (%) is shown in **Table 1**.

**Table 1:** Compositions of camel milk (%) (14).

Species	Fat	Protein	Lactose	Ash	Total Solids
Camel	4.9	3.7	5.1	0.7	14.4
Cow	4.5	3.8	4.9	0.72	13.9
Buffalo	7.6	3.8	4.9	0.78	17
Ewe	5.3	5.5	4.6	0.9	16.3
Goat	3.5	3.1	4.6	0.79	12
Mare	1.6	2.7	6.1	0.51	11
Ass	1.2	1.7	6.9	0.45	10.2
Reindeer	18	11	1.5	–	33
Elephant	15.1	4.9	3.4	0.76	26.9
Women breast milk	4.5	1.1	6.8	0.2	12.6

Maintenance of CM quality is associated with the enzymes aspartate aminotransferase (AST), alanine aminotransferase (ALT), gamma glutamyl transferase (γ-GT), acid phosphatase (ACP), alkaline phosphatase (ALP) and lactate dehydrogenase (LDH). In addition, CM lysozyme proteins, including Lf, lactoperoxidase and PGRP increase the half-life of CM.

### Protein

Proteins are an important component of CM and contribute significantly to its nutritional value. The proteins present in CM are diverse and differ in both composition and therapeutic properties (15). CM contains 3.73 - 3.89% total protein, which is a mixture of casein and whey protein (16). The most important protein group in CM is casein (52-87% of total protein), which consists of four main sub-types: α_s1_ casein (22%), α_s2_ casein (9.5%), β-casein (65%), and κ-casein (3.5%) (12, 17). The α_s1_ casein complex (lacking in human milk) is a major cause of milk protein allergies and is much more abundant in cow and buffalo milk (18) compared to CM.

Whey protein is 20 to 25% of the total protein of CM (19) and includes α-lactalbumin (α-La), serum albumin, lysozyme, Lf, peptide glycan detection proteins, lactoperoxidase and Ig. Beta-lactoglobulin (β-Lg) is the main whey protein (55%) and α-La is the second most abundant (20%) in cow milk. In camel and human milk, α-La is the major component of whey protein (20). In addition, CM, like human milk, lacks β-Lg, which is one of the most crucial proteins behind milk allergies. Like cow milk, the major whey protein in buffalo, sheep and goat milk is β-Lg (20).

CM also has insulin/insulin-like proteins that regulate the activity of immune response β cells, as well as having a therapeutic effect on blood sugar.

### Bioactive Native Proteins

Various immunoglobulins, including IgM, IgG, IgA and even IgD are found in CM (21). Immunoglobulins protect against bacterial and viral infections, including tuberculosis (22). Immunoglobulins that are involved in fighting against infections are among the native proteins of CM but not abundantly present in the milk of other mammalian species such as goats, buffalo, cattle, sheep and humans. CM contains the highest level of IgG (1.64 mg/ml) (23). The immunoglobulins in CM are one-tenth the size of human antibodies and are easily absorbed from the intestine into the general circulation. Therefore, CM immunoglobulins can reach tissues and cells that human immunoglobulins cannot (24).

One of the glycoproteins in CM is Lf (0.22 mg/ml), which is a lot higher compared to goat, sheep, buffalo and cow milk (25). Lf has been demonstrated to have antioxidant properties and is also used to transport and store iron. Lf levels decrease during the period of milking (26) and the highest levels are observed 2 days postpartum. Lf is a protective protein of CM as it prevents the proliferation of pathogens and microbes (27).

### LIPIDS

The fat content of CM is 2.6 to 3.2% (28). The white color of CM is due to the lower concentration of carotene in fat (29). CM fat contains lower concentrations of short-chain fatty acids (30) and higher concentrations of long-chain fatty acids (31) compared to cow milk, and this also raises the melting and freezing point. CM also contains more cholesterol (34.5 mg/100 g) than cow milk (25.63 mg/100 g) (32).

The main source of energy in milk is fat, and fat molecules range in size from 0.1-18 micrometers in diameter (33). Lipids include phospholipids (25%), cerebrosides (3%) and cholesterol (2%), and make up 30% of the cell membrane. Proteins make up 70% of the remaining membrane. Each fat unit consists of a triglyceride nucleus and a natural biological membrane that contains cholesterol, enzymes, glycoproteins, and glycolipids (34).

CM contains conjugated linoleic acid (CLA) that improves the plasma LDH/high-density lipoprotein (HDL) ratio by lowering the levels of triglycerides. In bone tissue, the production of anti-inflammatory cytokines and changes to the concentration of insulin-like growth factor can be stimulated by CLA and results in bone formation (35), muscle mass increased (36, 37) and subcutaneous fat decreased (38).

### Minerals And Vitamins

Calcium, phosphorus, sodium, potassium, chloride, iodine, magnesium and a small amount of iron are among the minerals that are found in CM (15, 39), which are expressed as whole ash (0.82-0.85%) (24). The amount of iron, zinc and copper in CM is higher than in cow milk (40).

Factors such as breed, nutrition, water consumption and analytical methods can change the mineral content of CM (41, 42). Camels usually graze on halophilic plants rich in chloride such as Atriplex, Salosa and Acacia (12) to meet their physiological salt needs (43).

Water-soluble and fat-soluble vitamins are present in CM. CM contains vitamins C, A, E, D and group B (41, 44). The concentration of vitamin C in CM (34.16 mg/L) is 2 to 3 times higher than in cow milk (44) and 6 times higher than in human milk (15). The vitamin C content of CM causes a low pH and as a result, it makes the milk stable and it can be kept fresh for a longer time. Vitamin C has strong antioxidant activity in CM (45). CM colostrum contains more vitamins A, E and B1 than adult CM (29). The composition of camel milk is summarized in **Table 1**.

## Results

### Anti-Inflammatory Effects

Antimicrobial, antioxidant, antihypertensive, anti-inflammatory and immune system modulatory activities of CM are linked to its bioactive components (46) such as, vitamins C, A, and B2. CM has anti-inflammatory effects (46) in some infectious diseases, including infection induced by hepatitis C virus (46).

The main components of CM, such as lysozyme, Lf and lactoperoxidase are important in the treatment of some inflammatory diseases, including hepatitis, allergies, lactose intolerance and liver damage caused by alcohol in some parts of the world. In addition, the therapeutic benefits of CM in inflammatory diseases may relate to CM antibodies that regulate the immune system (47). Lf in CM has potent antimicrobial and anti-inflammatory activity, this includes improvement of maturation and function of lymphocytes (27). One of the most important immuno-modulatory cytokines is tumor necrosis factor-α (TNF-a), which enhances the inflammatory response by stimulating the production of reactive oxygen species (ROS), arachidonic acid metabolites, proteases, and some cytokines (48). When inflammatory cells (neutrophils, macrophages, and lymphocytes) are activated, they create oxidative stress by producing ROS such as superoxide anions, hydrogen peroxide, and hydroxyl radicals (48). CM consumption reduces oxidative stress produced by the immune system’s anti-inflammatory response. This section provides details of key animal and clinical studies of the anti-inflammatory properties and mechanisms of CM. The anti-inflammatory effects of CM are shown in **Table 2**.

**Table 2:** Anti-inflammatory effects of camel milk.

Agent	Effects	Model of study	Doses	Ref.
CM	Inhibited the volume of edema	Rats, paw edema	33 ml/kg	(49)
CM	Reduced the index of osteoarthritis, paw edema and gait score, increased IL-10 serum	Rats, RA	10 ml/kg orally, 3 weeks	(50)
CM	Decreased inflammatory angiogenesis, the collagen deposition and VEGF levels	Albino Swiss mice	25-100 mg/kg/day, 14 days	(51)
CM	Reduced TGF-β1	Rats, T2D	35 ml/rat/day	(52)
Total lipid and fatty acids	Decreased IL-1β/18, regulated the expression of p50/p65 NF-κB subunits	*In vitro* diabetic inflammation	–	(53)
C whey protein	Reduced apoptosis, T and B cells distribution (spleen and thymus), increased AKT and IκB-α phosphorylation	T1D, Mice	100 mg/kg at 250 μl/day, 1 month	(54)
Un-denatured whey proteins	Decreased IFN-γ and increased IL-2	T1D rats	100 mg/kg/day, 5 weeks	(55)
C whey protein	Reduced IL-6, IL8, bloody stools, diarrhea, weight, and large intestine inflammation	Balb/c mice with colon cancer	–	(56)
CM	Improved leukocyte infestation, pathological changes, MPO and caspases-3 activities	Rats with TNBS-induced colitis	10 ml/kg	(57)
CM	Decreased CD8+ T cells, increased CD4+ T and CD44+ CD4+ cells	Mice model of ACC	2 g/kg/day in 200 µL	(58)
CM	Reduced ulcers number, ulcers length, ulcer index and the volume of gastric juice	Rats, gastric ulcers	5 ml/kg	(59)
CM	Reduced IL-1β, increased IL-10	C57BL/6J mice	0.4 ml/day, 14 days,	(60)
Fermented CM	Reduced CRP and IL-1ß	HFD-induced … in rats	–	(61)
CM	Reduced IL-6 expression	Liver injury, rats	100 ml/day	(62)
Lactic acid bacteria	Decreased in IL-6	Acute liver damage, mice	for 7 weeks	(63)
CM	Increased IL-10, DOPA and AChE, improved sensorimotor function and impaired memory	FNP-induced neurotoxicity in rats	2 ml/rat/day	(64)
CM	Reduced IL-1β in lung tissue and neutrophil infiltration	Rats, ARDS	10 mL/kg	(65)
CM	Inhibited MPO, IL-1β, IL-18 and MCP-1	A model of renal toxicity in rats	10 ml/kg p.o.	(66)
CM	Decreased renal inflammation	Cyclosporine-induced RI in rats	10 ml/kg, 3 weeks	(67)
CM	Inhibited single-cell chemotactic protein, hyaluronic acid and TGF-b1 serum levels	Hepatitis C infected patients	5 L/week, 2 month	(68)
CM	Decreased IL-4, increased IFN-γ	Chronic hepatitis Patients	–	(69)
CM	Decreased serum TARC	Double-blind in autism patients	500 ml/day	(70)

ACC, Acute and chronic colitis; AChE, Acetylcholinesterase; AKT, Protein kinase B; ARDS, Acute respiratory distress syndrome; CM, Camel milk; CRP, C reactive protein; DOPA, Dopamine; FNP, Fenpropathrin; IFN-γ, Interferon gamma; IκB-α, Nuclear factor of kappa light polypeptide gene enhancer in B-cells inhibitor-alpha; IL, Interlukin; MCP-1, Monocyte chemoattractant protein-1; MPO, Myeloperoxidase; NF-κB, Nuclear factor-kappa B; RA, Rheumatoid arthritis; TARC, Thymus and activation‐regulated chemokine; TGF-β1, Transforming growth factor-beta1; TNBS, Trinitrobenzene sulfonic acid; T1D, Type 1 diabetes; T2D, Type 2 diabetes; VEGF, Vascular endothelial growth factor; HFD, High-fat diet; RI, Renal injury.

#### Anti-Inflammatory Effects of CM, Animal Studies

Administration of CM (33 ml/kg) in rats, inhibited the inflammation and paw edema caused by injection of acetic acid. In a rat model of rheumatoid arthritis (RA), the inflammatory inhibition effect of CM was shown after administration of CM (10 ml/kg orally for 3 weeks) demonstrated by a reduction of the index of osteoarthritis, paw edema and gait score, along with the migration of inflammatory cells to the dorsal sac and increased interleukin (IL)-10 in rat serum (50). This study shows the potential of CM as a supplement in the management of RA (50). The anti-inflammatory effect of CM (25, 50 and 100 mg/kg/day for 14 days) in a angiogenesis mouse model was demonstrated by a reduction of collagen deposition, decreased vascular endothelial growth factor (VEGF) levels, reduced vascular (content Hb) and macrophage uptake (NAG activity) and IL-1β, IL-6, and IL-17 levels (51). Therefore, inflammatory angiogenesis was inhibited by down-regulation of pro-angiogenic and pro-inflammatory cytokines when mice were treated with CM. CM decreased MPO and NAG activities. In addition, CM reduced the wet weight of the implant by reducing the density of fibrovascular tissue induced by the sponge matrix, and by reducing the number of fibroblasts and mononuclear cells accompanied by less arteries (51).

Treatment of type 2 diabetic rats induced by intraperitoneal (i.p.) injection (58) of streptozotocin (STZ) with CM (35 ml/rat/day), reduced the level of transforming growth factor beta 1 (TGF-β1) which indicate anti-inflammatory activities. Production of pro-inflammatory cytokines (TNF-α) and increased IL-1β/18 ratio in diabetic inflammation in differentiated ThP1 cells were significantly decreased by application of total lipids (TL) and total fatty acids (TFA) derived from CM. The expression of p50/p65 sub-units of NF-κB and nucleotide-binding oligomerization domain-like receptor family pyrin domain containing-3 (NLRP3) were regulated, but the expression of regulatory cytokines IL-10 and IL-1 receptor antagonist (IL-1Ra) and cluster of differentiation 163 (CD163)-shifting cells towards an M2 macrophage phenotype were increased by TL. These findings indicate that CM can modulate of the expression of inflammatory regulators such that inflammation is reduced (53). Treatment with camel whey protein (CWP, 100 mg/kg at 250 μl/day for 1 month) of mice with type 1 diabetes (T1D), decreased apoptosis and the distribution of T cells and B cells in the spleen and thymus, but phosphorylation of protein kinase B (AKT) and IκB-α were increased by CWP. Treatment with CM denatured whey proteins (DMWP, 100 mg/kg for five weeks) on STZ-induced T1D rats decreased mRNA expressions of interferon gamma (IFN-γ) but increased IL-2 and their protein levels (55).

In Balb/c mice with colon cancer, CWP treatment reduced the expression of the inflammatory cytokines, IL-6 and IL-8 genes in colon tissue, reduced bloody stools and diarrhea, increased weight and reduced inflammation of the large intestine, thereby reducing the early stages of colorectal cancer (CRC) symptoms. The proliferation and differentiation of proinflammatory cells are decreased by CM, and CM regulated the number of anti-inflammatory cells in the rat colon and hence reduced the symptoms of colitis (52). In addition, colitis induced by chemical stimuli was also reduced by CWP. CM whey increased the regulation of the IL-10 gene, which can lead to a significant reduction in the number of lumps and symptoms of colitis (56). Further, pre-treatment of CM (10 ml/kg, orally) of rats with colitis induced by trinitrobenzene sulfonic acid (TNBS), suppressed the severity of intestinal damage, improved the weight/length ratio of the large intestine, leukocyte infestation, macroscopic damage, histopathological changes, myeloperoxidase activity and the activity of apoptotic marker (caspases-3) (57) In IBD, mainly leukocyte uptake by TNF-α and ROS was observed through the increased expression of P-selectin, ICAM and MAdCAM-1 adhesive molecules in the colonic mucosa. Inhibition of TNF-α and oxidative stress by CM reduced the leukocyte influx (71, 72). In addition, in a mouse model of sulfate (DSS)-induced acute and chronic colitis, treatment with CM (2 g/kg B.W in 200 µL DDW/day) reduced DSS-induced damage to the colon mucosa and immune cell imbalance (73). Moreover, administration of CM decreased the percentage of CD8+ T cells but increased CD4+ T and CD44+ CD4+ cells. Overall, CM-treated animals had a lower disease activity index (DAI) and histopathological score compared to untreated mice (58). These results indicate that CM could be considered as a complementary therapeutic approach for inflammatory bowel disease (IBD). Direct inhibition of infection by an attack of pathogens occurs with Lf and CM lysozyme, which enhances the local Th1 response. Therefore, the host’s important defense approach against bacterial infections by the function of the immune regulator is strengthened (54, 74, 75).

Treatment of ethanol and aspirin-induced gastric ulcers with CM (5 ml/kg B.W) reduced the number of ulcers, average length of ulcers, ulcer index and the volume of gastric juice in comparison to control animals, demonstrating the protective effects of CM against gastric ulcers in rats (59). The anti-inflammatory effects of CM has also been demonstrated in radiation-induced intestinal damage in C57BL/6J mice. The results showed that treatment with CM (0.2 ml twice daily for 14 days by gavage) reduced serum levels of IL-1β, reduced the radiation injury score (RIS) and increased IL-10 levels. Furthermore, intervention with CM regulated the intestinal protein expression of the HMGB1/toll-like receptor-4 (TLR) pathway, TLR4, nuclear factor kappa B (NF-κB) and HMGB1). In addition, CM protected animals against jejunum damage caused by radiation by regulating the inflammatory signaling pathway: HMGB1/TLR4/NF-kB/MyD88 (60), Activation and release of HMGB1 occur through activation of immune cells and inactivation of necrotic and damaged cells, respectively. As an inflammatory agent, HMGB1 exerts a set of cellular regulatory functions (including maturation, proliferation, motility, inflammation, survival, and cell death) after release into the extracellular space and interacting with a large panel of cell surface receptors (76–79). Furthermore, HMGB1 secretes direct proinflammatory cytokines by binding directly to TLR4. The effect of fermented CM (FCM) on inflammation associated with a high-fat diet in albino rats reduced IL-1ß and C reactive protein (CRP) (61).

Pre-treatment with CM (100 ml/day) of rats with liver injury induced by *Escherichia coli* (*E. coli*) and *Staphylococcus aureus* (*S. aureus*), before injection of *E. coli* and *S. aureus*, reduced IL-6 expression, lipid peroxidation, antioxidant defense system activity and immune cytokines (62). Administration of six strains of lactic acid bacteria (LAB) from CM for 7 weeks in animals with acute liver damage caused by LPS/D-galactosamine (D-GalN), significantly decreased IL-6 levels compared to controls. In this study, 107 LAB strains were identified and isolated from Mongolian CM products. Thirty-six *Lactococcus bacteria* belonging to 8 different species and 71 *Lactobacillus bacteria* belonging to 9 different species were identified and six LAB strains were studied. The studied bacterial species were WXD5 (*Lactobacillus (L.) paracasei subsp. paracasei*), WXD30 (*L. casei*), WXD55 (*L. plantarum subsp. argentoratenis*), WXD100 (*L. plantarum subsp. argentoratenis*), WXD101 (*L. plantarum subsp. argentoratenis*), WXD106 (*L. plantarum subsp. argentoratenis*) (63). The anti-inflammatory activity of CM was also effective in a neurotoxin model with fenpropathrin (FNP) in rats. In this study, treatment with CM (2 ml/rat/day) increased IL-10, improved sensorimotor function, impaired memory and exploration, and increased dopamine (DOPA) and acetylcholinesterase (AChE) levels (64). By protecting areas of the brain against FNP-induced damage, CM enhanced sensorimotor function (80). The presence of high amounts of copper, zinc, magnesium, vitamins E and C (81) has led to the beneficial role of CM in maintaining sensory-motor balance (82, 83).

In rats with lipopolysaccharide (LPS)-induced acute respiratory distress syndrome (ARDS), CM (10 ml/kg of BW) showed anti-inflammatory effects by reducing the amount of pro-inflammatory cytokines, IL-10 and IL-1β in the lung tissue, mitogen-activated protein kinase signaling pathways, alveolar wall thickness, lung injury scores, interstitial and intra-alveolar edema. Also, the LPS-induced increase in neutrophil infiltration was significantly reduced in CM-treated animals compared to controls (84).

Interestingly, the inhibitory effect of CM on carcinogenicity is mainly due to its derived exosomes. In addition, in tumor tissues, CM and especially its exosomes reduced the genes associated with inflammation including IL1β and NFκB, which express the anti-inflammatory effects of CM and its exosomes (85).

In a rat model of renal toxicity, administration of CM (10 ml/kg, p.o.), suppressed renal inflammation by inhibiting myeloperoxidase (MPO), IL-1β, IL-18 and monocyte chemoattractant protein-1 (MCP-1) (66). Moreover, administration of CM (10 ml/kg/day of CM for 3 weeks) on cyclosporine-induced renal injury in male Wistar rats, markedly reduced the cancers, indicated by improving serum creatinine and blood urea nitrogen (BUN) levels, as well as kidney injury molecule-1 (KIM-1). As a result, CM decreased renal inflammation by the anti-inflammatory pathway of p38/extracellular signal-regulated kinases (ERK)/c, Jun N-terminal kinases (JNK) and mitogen-activated protein kinases (MAPK) (67).

#### Anti-Inflammatory Effects of CM, Clinical Studies

Interestingly, treatment of patients with hepatitis C virus with 5 litres of fresh CM/week for 2 months, reduced serum levels of pro-inflammatory markers including single-cell chemotactic protein, hyaluronic acid, MCP-1 and TGF-β1. These results indicated the therapeutic effect of CM on naturopathic condition (68).

In a double-blind randomized clinical trial (RCT), the effect of CM (raw, boiled, 500 ml/day) on patients with autism was evaluated (70). Thymus and activation‐regulated chemokine (TARC) serum levels significantly decreased (P = 0.004) in boiled CM and in raw CM group (P = 0.001). Furthermore, the childhood autism rating scale (CARS) score were significant improved (P = 0.04) only in raw CM group (70).

### Antioxidant Effects

To maintain cellular homeostasis, biological systems produce ROS which mediate the synthesis of various substances including hydroxyl radicals, NO radicals, superoxide anions, and peroxyl radicals (86). While excessive production of free radicals may lead to damage to deoxyribonucleic acid (DNA), proteins and lipids (87), the body’s natural antioxidant system is able to eliminate these free radicals (88). The exogenous antioxidant property of CM was demonstrated in several studies by reducing oxidative stress (27, 89, 90). It is proposed that the higher antioxidant activity of CM is likely to be due to the 6.7 times more vitamin C in fresh CM than fresh cow milk, and in addition to the presence of other antioxidant components such as caseins, LABs, bioactive peptides, whey proteins, and especially lactoferrin (91, 92). The antioxidant effects of CM are shown in **Table 3**.

**Table 3:** Antioxidant effects of Camel milk.

Agent	Effects	Model of study	Doses	Reference
CM	Increased ACE inhibitor activity	TEAC	–	(93)
CM casein	Inhibited ABTS, DPPH and FRAP activities	ABTS, DPPH and FRAP method	–	(94)
CM non-fat powder	Increased the antioxidant activity	ABTS, DPPH and FRAP method	–	(95)
CM	Increased SOD and CAT in liver homogenates	CYP-induced leukopenia in mice	2 ml/day for 10 days	(96)
CM	Reduced lipid peroxide and NO, increased GSH and TAC in serum	RA model of rat	10 ml/kg, 3 weeks	(50)
Fermented CM	Improved the activities of SOD, CAT, GPx	Mice heart tissue exposed to CCl4	100 mg/kg	(10)
CM and its exosomes	Reduced MDA and iNOS, increased SOD, CAT and GPx activities	Rats and MCF7 breast cancer cells	1 ml/rat, orally	(85)
CM protein hydrolysates	Reduced MDA and GSH, improved SOD activity	Diabetic rats	100,-1000 mg/kg, 8 weeks	(97)
CM peptide	Increased SOD, CAT and GSH, decreased MDA	Diabetic rats	25 mg/kg, 7 days	(74)
CM	Reduced MDA, increased SOD and CAT	Rabbits model of diabetes	7 ml/kg, 4 weeks	(98)
CM	Decreased MDA, increased CAT, GR and SOD	Rat model of diabetes	–	(47)
CM	Increased SOD, CAT and GSH	STZ -induced DM in rats	50 ml/day, 8 weeks	(99)
CM protein	Decreased ROS and ATF-3 expression	Mice model of TID	100 mg/kg at 250 μl/day, 1 month	(54)
CM	Reduced MDA, increased GST and SOD	Rats model of liver disease	100 ml/day	(62)
CM	Inhibited MDA and MPO, restructured SOD and GST activities	GM-induced liver damage in rats	5 mL/rat/day	(100)
Fermented CM	Increased SOD, GPx, CAT and GSH in the liver, decreased MDA	CCl4 liver damage in mice	–	(101)
CM	Reduced hepatic MDA and increased TAC	Alcohol-induced hepatotoxicity, rats	2 ml/day	(102)
CM	Increased GSH and CAT, decreased MDA	Rats with NAFLD	50 ml/day, for 8 weeks	(103)
C Lactoferrin	Decreased NO	HCT-116 colon cancer cells	–	(27)
CM	Reduced lipid peroxides and NO	Rats model of IBD	20 ml/kg/day gavage	(57)
CM	Increased SOD and GSH, decreased MDA	C57BL/6J mice	0.4 ml/day, 14 days	(60)
CM	Reduced NO, MDA, MPO and caspase-3	FNP-induced neurotoxicity in rats	2ml/day	(64)
CM	Reduced MDA, MPO and TAC in lung tissue	Rats model of ARDS	10 mL/kg/day	(65)
CM	Suppressed oxidative stress, improved GSH, SOD, GPx and TAC	5-FU-induced renal toxicity in rats	10 ml/kg p.o.	(66)
CM	Reduced MPO activity	Cyclosporine-induced kidney damage in rats	10 ml/kg/day, 3 weeks, gavage	(67)
CM	Increased SOD and CAT activities	CdCl_2_-induced toxicity in rats	2 ml, 21 days	(84)
CM	Increased SOD, CAT and GSH activities	CdCl_2_-induced HMiA in rats	–	(104)
CM	Reduced TBARS and HP, increased GSH, SOD and CAT activities	AlCl3-induced oxidative stress rat	1 ml, 30 days	(105)
CM	Increased in plasma levels of GSH, SOD, decreased MPO	Double-blind RCT in ASD patients	500 ml/day, 2 weeks	(106)

ABTS, 3-ethylbenzthiazoline-6-sulphonic acid; AlCl_3_, Aluminum chloride; ARDS, Acute respiratory distress syndrome; ASD, Autism spectrum disorder; CM, Camel milk; CCl4, Carbon tetrachloride; CYP, Cyclophosphamide; DN, Diabetic nephropathy; DPPH, 2,2-diphenyl-1-picryl-hydrazyl-hydrate; FRAP, Ferric reducing antioxidant power; GM, Gentamicin; GPx, Glutathione peroxidase; GR, Glutathione reductase; GSH, Glutathione; GST, Glutathione transferase; HP, Hydroperoxide; IBD, Inflammatory bowel disease; iNOS, Inducible nitric oxide synthase; MDA, Malondialdehyde; MPO, Myeloperoxidase; NAFLD, Nonalcoholic fatty liver disease; NO, Nitric oxide; NOX-1, Nicotinamide adenine dinucleotide phosphate oxidase; RA, Rheumatoid arthritis; SOD, Superoxide dismutase; TAC, Total antioxidant capacity; TNBS, Trinitrobenzene sulfonic acid; CdCl_2_, Cadmium Chloride; HMA, Hypocromic microcytic anemia; TEAC, Trolox equivalent antioxidant capacity.

#### Antioxidant Effects of CM, Animal Studies

The antioxidant activity of CM was evaluated using the Trolox equivalent antioxidant capacity scale (TEAC). The antioxidant and the angiotensin-converting enzyme (ACE) inhibitor activity of camel total casein and camel β-caseins, increased after enzymatic hydrolysis. In fact, the produced peptides start to act as natural antioxidants and ACE-inhibitors when CM is consumed and digested. CM also possesses antioxidant and protective activity by preventing damage to skin (dryness and wrinkles) by slowing down the production of free radicals (93). Moreover, the antioxidant activity of casein hydrolysates of CM has significant inhibitory activity in ABTS, 2,2-diphenyl-1-picryl-hydrazyl-hydrate (DPPH) and ferric reducing antioxidant power (FRAP) assays (94). Hydrolysis conditions and enzymes used affect the DPPH radical scavenging activity of the whey protein hydrolysate (95). The antioxidant activity of CM non-fat powder (NFCM) using ABTS, DPPH and FRAP methods, showed remarkable antioxidant activity (96).

In addition, in mice with cyclophosphamide (CYP)-induced leukopenia, CM (1 ml, twice daily for 10 days) increased the levels of superoxide dismutase (SOD) and catalase (CAT) in liver homogenates, acting as an immune system booster (107). Interestingly, the neutrophil function was improved in older mice by CM protein (108). In a RA rat model, CM (10 ml/kg orally for 3 weeks) decreased the lipid peroxide and NO production and increased serum levels of glutathione (GSH) and TACh (50).

The cardio preventive potential of FCM by *Lactococcus lactis subsp cremoris* (FCM-LLC, 100 mg/Kg/day, for 15 days) against the toxic effects of acute exposure of heart tissues of mice to carbon tetrachloride (CCl4), improved SOD, CAT and GPx activities, oxidative stress and attenuated cardiac toxicity (109). Due to the presence of high amounts of antioxidant compounds including oligosaccharides, vitamins, bioactive peptides and conjugated linoleic acid, fermented CM showed antioxidant effects on heart tissue (110). Further, high levels of magnesium in fermented CM (111) plays an important role in GSH biosynthesis (112) and therefore reduces oxidative stress. In addition, Vitamin C prevents cell damage by scavenging free radicals (113).

Both an *in vitro* and *in vivo* study on MCF7-induced breast cancer cells in rat showed CM treatment reduced malondialdehyde (MDA) levels and inducible nitric oxide synthase (iNOS) gene expression. In addition, SOD, CAT and GPX activities were increased (85) leading to significant inhibition of oxidative stress.

Furthermore, the effect of CM protein hydrolysate treatment (CMPH, 100, 500 and 1000 mg/kg BW, for 8 weeks) in STZ-induced diabetic rats was associated with inhibition of oxidative stress by reducing the content of MDA and GSH levels and a significant improvement in SOD activity (97). The antioxidant effect of peptides extracted from CM (CMP, 25 mg/kg B.W for 7 days) on diabetic rats, significantly increased the levels of SOD, CAT and GSH but decreased the level of lipid oxidation. The antioxidant potential of CMP has been proven based on its ability to restore the natural state of redox with a single dose, but further investigation is needed to support this claim (74). Administration of CM (7 ml/kg for four weeks) compared with insulin therapy in experimental diabetes in rabbits, significantly reduced MDA serum levels compared to the insulin group. In addition, the levels of SOD and CAT in the CM-treated group were significantly higher than the untreated group (98). High levels of vitamin C and minerals including copper, potassium, sodium, zinc, iron and magnesium in CM resulted in a strong antioxidant effects of CM in the fight against free radicals (114, 115). Furthermore, in a rat model of diabetes, treatment with CM for two months, decreased MDA levels and increased CAT, glutathione reductase (GR) and SOD levels indicating the antioxidant activity of CM (47). Deactivation of reactive oxygen species by CM casein was observed by scavenging free radicals (116). In rats with STZ-induced diabetic nephropathy, treatment with CM (50 ml/day, for 8 weeks), increased SOD and CAT expression, CAT activity and GSH levels, as well as reducing fat peroxidation. Therefore, CM showed protective effects against diabetic nephropathy by its antioxidant effects (99). Administration of CWP to mice with T1D (100 mg/kg B.W at 250 μl/day for 1 month) decreased ROS and ATF-3 expression (54).

In rats with liver disease due to experimental infection with *E. coli* and *Staphylococcus aureus*, treatment with CM (100 ml/day) reduced MDA levels but increased the expression of GST and SOD (62). Similarly, in gentamicin (GM)-induced liver damage in rats, administration of CM (5 ml/rat/day), inhibited MDA formation and MPO activity, and reconstitutes SOD and glutathione transferase (GST) activities. These results showed a protective effect of CM against GM-induced hepatic damage by inhibiting oxidative stress (100). The protective effect of FCM on CCL4-induced liver damage was observed by enhancement of SOD, glutathione peroxidase (GPx), CAT and GSH levels in the liver, while MDA levels declined (101). Administration of CM (2 ml daily) in alcohol-induced hepatotoxicity in male rats, significantly reduced hepatic MDA and increased total antioxidant capacity (TAC). Therefore, the antioxidant properties, and possible chelating effects on free radicals, of CM countered some of the physiological harms caused by ethanol-induced hepatotoxicity (102). CM treatment (50 ml/day, for eight weeks) of male Wistar rats with nonalcoholic fatty liver disease (NAFLD), increased GSH levels and CAT activity but decreased MDA levels (103).

Application of camel Lf to HCT-116 colon cancer cells resulted in the lowest amount of FRAP, the least inhibitory effect on DPPH radicals and a significant increase in NO capacity inhibition, all in a dose-dependent manner (27). In TNBS-induced colitis in rats, administration of CM (10 ml/kg by gavage) reduced serum levels of NO and MDA and increased the levels of GSH and TAC, ultimately strengthening the antioxidant defense system (57). Administration of CM (20 ml/kg/day by oral gavage) in rats with IBD-suppressed intestinal damage and reduced the levels the cytokines, TNF-α and IL-10 in the large intestine. In addition, CM strengthened antioxidant defense by enhancing colon GSH and suppressing oxidative stress by reducing lipid peroxides and NO (57) Also, mice with radiation-induced intestinal damage had increased SOD and GSH levels and decreased MDA serum levels when treated with CM (60). The antioxidant properties of CM are due to the high content of vitamins C and E along with selenium, zinc and other trace elements (106, 117).

The effects of CM (2 ml/day) on FNP-induced neurotoxicity in rats, improved DOPA and AChE levels, decreased NO, MDA, MPO, Caspase-3, and TNF-α levels but increased IL-10, TAC, and Bcl-2 levels. Neurodegenerative changes in the hippocampus induced by FNP was also improved CM (64). CM magnesium reduces oxidative stress and increase the absorption of vitamins E and C.thus CM has antioxidant activity (65). CM treatment (10ml/kg) in LPS-induced ARDS in rats reduced the amount of MD, MPO and TAC in the lung tissues (84).

In rats with renal toxicity induced by 5-FU, CM administration (10 ml/kg orally) suppressed oxidative stress by improving GSH, SOD, GPx and TAC. Therefore, CM showed a protective effect against kidney toxicity by inhibiting ROS activity (66). Treatment with CM (10 ml/kg/day; for 3 weeks by gavage) in cyclosporine-induced kidney damage in male Wistar rats, reduced MPO activity but increased the reduced/oxidized ratio of GSH and TAC (67). Further, in albino white rats with cadmium chloride-induced toxicity, daily consumption of CM (2 ml of fresh CM for 21 days) increased SOD and CAT activities, indicating a protective effect of CM against the toxicity caused by cadmium chloride (104). Similarly, in CdCl_2_-induced HMiA in rats, CM increased SOD and CAT activities as well as GSH concentration. In addition, the production of free radicals was reduced, revealing the protective effect of CM against oxidative stress (105). In male rats with oxidative stress induced by aluminum chloride (AlCl_3_) and lipid peroxidation in the testes, treatment with CM (1 ml of fresh CM for 30 days) significantly reduced lipid peroxidation biomarkers (TBARS and HP) and increased GSH, SOD and CAT activities in the testes (106).

#### Antioxidant Effects of CM, Clinical Studies

In a RCT, the effect of CM (average 500 ml, daily for 2 weeks) in 60 subjects with autism spectrum disorder (ASD) caused a significant increase in serum levels of GST and SOD, but decreased MPO. Therefore, CM as a regulator of antioxidant enzymes showed reduced oxidative stress in ASD (118).

### Immunomodulatory Effects

The main function of the immune system is to protect against pathogens including bacteria, viruses, fungi and toxins. Among the cells involved in the defense system are neutrophils, monocytes, macrophages and lymphocytes. The acquired immune system is able to increase the immune response by creating immunological memory in subsequent encounters with pathogens (119).

CM has high levels of protective proteins such as lysozyme, IgG and secretory IgA compared to cow’s milk (19). Insulin-like activity has also been observed in CM, which acts by regulating and modulating functions of ß cells. Therefore, CM showed immunomodulatory in various immune-mediated disorders. The immunomodulatory effects of CM are shown in **Table 4**.

**Table 4:** Immunomodulatory effects of camel milk.

Agent	Effects	Model of study	Doses	Reference
CM	Reduced TNF-α	Male offspring of pregnant female rat model induced by VPA	2 ml/p.o	(14)
CM	Reduced TNF-α in serum	Rats, adjuvant-induced arthritis	10 ml/kg, 3 weeks	(50)
CM	Reduced TNF-α	Alcohol-induced hepatotoxicity in male rats	2 ml	(102)
CM	Reduced TNF-α and TGF-β	Angiogenesis model in mice	25-100 mg/kg/day, 14 days	(51)
CM	Reduced TNF-α	Rat model of T2D	35 ml/day	(52)
Total lipid and fatty acids	Decreased TNF-α	*In vitro* diabetic inflammation	–	(53)
C whey protein	Decreased IL-1β, IL-6, increased IL-4	Mice model of T1D	100 mg/kg B.W at 250 μl/d, 1 month	(54)
Un-denatured whey proteins	Increased B cells, mitogen-stimulated lymphocyte proliferation, reduced T cells and TNF-α	Mice model of T1D,	100 mg/kg in 250 μl/day, 5 weeks	(55)
Fermented CM	Reduced TNF-α	High-fat diet in twenty-eight albino rats	1 ml	(61)
Lactic acid bacteria	Reduced TNF-α	LPS/D-GalN in mice	–	(63)
CM	Reduced TNF-α in the colon	TNBS-induced colitis in rats	10 ml/kg, gavage	(57)
CM	Reduced TNF-a	ACC-induced DSS in mice	–	(58)
CM whey	Reduced IFN-γ and IL8 in colon tissue	Balb/c CRC mice	–	(56)
CM	Reduced TNF-α	Radiation-induced intestinal damage in mice	0.2 ml, 14 days	(60)
CM	Reduced TNF-α	FNP in rats	2ml/day	(64)
CM	Reduced TNF-α	ARDS rats	10 ml/kg	(65)
CM	Inhibited TNF-α	Renal toxicity in rats	–	(66)
CM	Reduced TNF-α, MCP-1, IL-1β and IL-18	Cyclosporine-induced kidney damage, rats	10 ml/kg/day, 3 weeks	(67)
CM	Reduced TNF-a	Clinical study, 25 patients	5 L/week, 2 months	(68)

ACC, Acute and chronic colitis; CM, Camel milk; DSS, Sodium dextran sulfate; IFN-γ, Interferon gamma; IL, Interleukin; MCP-1, Monocyte chemoattractant protein-1; T1D, Type 1 diabetes; T2D, Type 2 diabetes; TGF-β1, Transforming growth factor-beta1; TNF-α, Tumor Necrosis Factor-alpha; VPA, Valproic acid.

#### Immunomodulatory Effects of CM, Animal Studies

Several experimental studies showed immunomodulatory effects of CM. In adjuvant-induced arthritis and models of air sac edema in rats, treatment with 10 ml/kg of CM for three weeks, reduced serum TNF-α, claw edema and the osteoarthritis index (50). Zinc modulated the immune response by inhibiting interleukins, prostaglandins, and other inflammatory cytokines (120, 121). CM contains higher amounts of zinc than other species’s milk (73), and the presence of Ig in CM regulates innate immunity which both contribute to the immunomodulatory effect of CM (49). Treatment with CM in alcohol-induced hepatotoxicity in male rats caused a significant reduction in hepatic TNF-γ, demonstrating a possible protective effect of CM on liver tissue damaged by alcohol toxicity (103). In a sponge implant angiogenesis model in albino Swiss mice, administration of CM (25, 50 and 100 mg/kg/day for 14 days) reduced TNF-α and TGF-β serum levels (51).

In rats with T2D, induced by STZ (40 mg/kg/day for four days), administration of CM (35 ml/day) reduced TNF-α levels, indicating the anti-diabetic effect of CM by its immunomodulatory activity (52). Furthermore, application of TL and TFA, in a model of diabetic inflammation in macrophages, decreased TNF-α and IL-1β/18 levels. In addition, expression of regulatory cytokines IL-10 and IL-1 receptor antagonist (IL-1Ra) were increased by TL and suggests CM lipids regulate immune function (53). In mice with T1D, CWP-treatment, decreased the levels of pro-inflammatory cytokines IL-1β and IL-6 but increased IL-4 levels (54). In STZ-induced T1D mice, treatment with DMWP (100 mg/dissolved in 250 μl/day for five weeks) significantly increased the number of B cells in the peripheral lymphoid organs. Treatment also reduced T cells in all zones, especially in the periarteriolar lymphoid sheaths (PALS) of the spleen sections of the diabetic rats. In addition, the DMWP-treated mice, had increased mitogen-stimulated lymphocyte proliferation (Con A or LPS) in their splenic follicles. Host cell proteins interact extensively with CM’ Ig and hence weakens the immune system and rescues β cells through an increase in regulatory cells (122). Treatment with DMWP decreased TNF-α mRNA expression. Overall, DMWP improves autoimmune function by regulating TNF-α and programmed cell death-receptor (Fas), suppressing self-activated T cells and improving pancreatic function. Suppression of T cell activation and promotion of cell survival by up-regulation of Cdc42 expression is confirmed, which maintains simple T cell homeostasis. Therefore, improvement in the condition of pancreatic β cells follows T cell homeostasis (55).

Similar effects on TNF-α by CM have also been reported in other disease models and tissues. For example, eating a high-fat diet can induce inflammation, but treatment with FCM reduced TNF-α levels, which led to a reduction in inflammation (61). Treatment with WP resulted in higher levels of CD28 expression and subsequent recovery of higher IL-2 and IFN-γ levels and replication capacity (55). The protective effect of six strains of CM LAB in mice suffering acute liver damage induced by LPS/D-GalN, was shown by a significant reduction of TNF-α levels (63). Treatment with CM in TNBS-induced colitis in rats also resulted in a reduced levels of colonic TNF-α and IL-10. The severity of intestinal damage was suppressed by CM and macroscopic damage, histopathological alterations, leukocyte influx, colon weight/length ratio and myeloperoxidase activity were improved (57). Lactoferrin (an anti-inflammatory protein component of CM) can inhibit the production of pro-inflammatory cytokines such as TNF-α, IL-1 and IL-6 in response to lipopolysaccharide activation. Moreover, inhibition of NF-κB activation following internalization of lactoferrin to monocytes could mechanically inhibit the production of pro-inflammatory cytokines (50). Similarly, mice with acute and chronic colitis, induced by DSS had reduced TNF-α, as well as inhibited Th1 and Th17 proliferation responses, when treated with CM. In addition, pathogens were attacked by CM Lf and lysozymes, thereby inhibiting infection due to the enhancement of the local Th1 response and function of the immune regulator (58). The results showed that CM, reduced the migration of inflammatory cells to the intestinal tract and arthritis index by reducing the concentration of TNF-α (123). In a colitis model in Balb/c CRC mice, the concomitant treatment with CM whey reduced the IFN-γ in colon tissue (56). Finally treatment with CM (0.2 ml for 14 days) on radiation-induced intestinal damage in mice reduced TNF-α levels (60).

Treatment with CM (2ml/day) of FNP in rats, reduced TNF-α. Therefore, CM can be considered as a biological protective agent against behavioural, neurological disorders caused by FNP through its effect on immune system (64). In the autistic behaviors of male offspring of pregnant female rat model induced by valproic acid (VPA), treatment with CM (2 ml orally), significantly reduced TNF-α expression. Therefore, CM could be considered as a possible treatment for autism by regulating immunomodulatory pathways (69).

In LPS-induced ARDS in rats, administration of 10 ml/kg of CM significantly reduced TNF-α in lung tissue and down regulated mitogen-activated protein kinase signaling pathways indicating the therapeutic effect of CM as a supplementary to treat ARDS (84). The effects of CM on reducing 5-FU-induced renal toxicity in rats, inhibited TNF-α and thus suppressed renal inflammation (66). The effects of CM on cyclosporine-induced kidney damage revealed that oral administration of CM (10 ml/kg/day for 3 weeks) caused a significant reduction in TNF-α, MCP-1, IL-1β and IL-18 (67).

#### Immunomodulatory Effects of CM, Clinical Studies

The reduction of TNF-α levels by CM has also been reported in human clinical trials. Administration of 5 liters of fresh CM per week for 2 months (drinking) in patients of HCV, with mild to moderate parenchymal complications and mild cirrhosis, reduced TNF-α level (68). In patients with chronic hepatitis B, that consuming CM for one year, increased the level of cytokine IFN-γ and Th1 cells and decreased the levels of IL-4 and Th2 cells. Therefore, CM enhances the immune response by regulating the expression of Th1/Th2 cytokines (14).

#### The Effects of Bioactive Peptide of CM

CM is more digestible due to chymotrypsin and the enzyme trypsin than cow milk. Higher levels of amino acid residues in α-lactalbumin in CM have higher antioxidant potential than the cow milk (124). CM protein was hydrolyzed using pepsin and pancreatin and three peptides were obtained with LEEQQQTEDEQQDQL, YLEELHRLNAGY and RGLHPVPQ sequences and the antioxidant properties of these peptides were investigated. The results showed an inhibitory activity of these three peptides on free radicals. In addition, the YY-11 peptide increased the expression of superoxide dismutase and catalase genes in HepG2 cells (125). Casein protein (CCP) and CM whey protein (CWP) peptides were hydrolyzed with pepsin and their antioxidant properties were investigated. P-CCP and P-CWP peptides significantly increased the tolerance of yeast cells to peroxide oxidative stress. Therefore, the bioactive peptides of caseins and CM whey proteins have significant antioxidant activity (126). Using a laboratory protocol that mimics gastrointestinal digestion, CM, colostrum and colostrum whey proteins were hydrolyzed by pepsin and pancreatin and their antioxidant properties were investigated. The results showed that bioactive components of CM and colostrum proteins increased antioxidant activity and angiotensin I-converting enzyme (ACE) inhibitors (127).

The antioxidant potential of CMPH was investigated *in vitro* and in real food model systems. Proteolytic enzymes such as alkalase, bromelain and papain were used for hydrolysis. CMPH increased the radical inhibition of DPPH and its antioxidant properties. Therefore, the breakdown of milk proteins to produce more active peptides with antioxidant potential, increased the activity of radical inhibition of DPPH in CM after hydrolysis (128). In rats infected with Schistosoma mansoni, administration of colostrum and CM induced immunomodulatory activity by inducing GST and IgG before and after infection, as well as stimulating specific immune responses (124).

Decreased glutathione due to overuse of paracetamol (PCM) and acetaminophen leads to hepatotoxicity. This hepatotoxicity increased mitochondrial dysfunction and oxidative stress. In addition, as the hepatotoxicity increases, exolastic glutamic transaminase (GOT) and serum pyruvic transaminase (GPT) enzymes enter the bloodstream. Treatment with CM reduced the levels of serum enzymes and thus maintained the integrity of the liver cell membrane against paracetamol-induced toxicity (124). The antioxidant activity and inhibition of ACE hydrolysed of camel whole casein and β-CN were evaluated using the TEAC. The results showed that the peptide fractions obtained from the hydrolysis of camel whole casein and camel β-CN increased the antioxidant and ACE inhibitor activities (93).

Enzymatic hydrolysis of camel and bovine WPs in an *in vitro* study showed significantly higher antioxidant activity of camel’s WPs and its hydrolysis than the bovine WPs and its hydrolysis. This may be due to differences in the amounts of antioxidant amino acid residues in their primary structures. The ability to chelate metal cations (Glu, Asp, Lys, Arg, and His), the ability to donate protons to free radicals (Trp, Phe, Tyr, His, and Cys) as well as the transfer of antioxidant activity of amino acid residues to proteins. Significant differences in the antioxidant activity of WPs/WP hydrolyzed camels and bovine have been reported (129). The effects of bioactive peptides of CM are shown in **Table 5**.

**Table 5:** Effects of bioactive peptide of camel milk.

Agent	Effects	Model of study	Reference
LEEQQQTEDEQQDQL, YLEELHRLNAGY and RGLHPVPQ	Inhibited free radicals, Increased SOD and CAT	HepG2 cells	(125)
P-CCP and P-CWP	Increased the tolerance of yeast cells to peroxide oxidative stress	*In vitro*	(126)
Bioactive components of CM and colostrum proteins	Increased antioxidant activity and ACE inhibitors	*In vitro*	(127)
CMPH	Increased the radical inhibition of DPPH and its antioxidant properties	*In vitro* and in real food model systems	(128)
Colostrum and CM	Stimulated specific immune responses	Rats infected with Schistosoma mansoni	(124)
CM	Reduced serum enzymes, integrity of the liver cell membrane	Paracetamol-induced toxicity	(124)
Camel whole casein and β-CN	Increased the antioxidant and ACE inhibitor activity	*In vitro*	(93)
WP	Increased antioxidant activity	*In vitro*	(129)

ACE, Angiotensin I-converting enzyme; β-CN, Beta-casein; CAT, Catalase; CM, Camel milk; CMPH, CM protein hydrolysis; SOD, Superoxide dismutase; WP, Whey protein.

## Conclusion

The anti-inflammatory effects of CM have been demonstrated in several studies in different inflammatory conditions such as paw edema, angiogenesis, diabetic, gastrointestinal inflammatory disorders including IBD, radiation-induced gastrointestinal inflammation, intestinal infection, gastric ulcers and colon cancer, neurotoxicity inflammation and renal inflammatory disorders, arthritis, IBD, inflammatory respiratory disorders including asthma, and lung injury induced by noxious agents such as LPS. The anti-inflammatory properties of CM and its bioactive compounds was achieved by their effect on various inflammatory cells and mediators. In rheumatoid arthritis, the anti-inflammatory effects of CM improve the symptoms. In clinical studies, CM showed anti-inflammatory effects by inhibiting serum levels of pro-inflammatory markers including single-cell chemotactic protein, hyaluronic acid, MCP-1, TGF-β1 and chemokines in patients with hepatitis C virus and autism.

Numerous studies indicate the antioxidant effects of CM by inhibiting ABTS, DPPH and FRAP. In addition, CM is able to increase the levels of ACE, GSH, SOD, GPx, TAC and CAT. The level of lipid peroxides, NO, MDA, MPO, caspase-3 and expression of iNOS gene were also decreased by CM. The antioxidant properties of CM and its derivatives were shown in various oxidative stress disorders including skin disorders, leukopenia, RA, oxidative stress of heart tissues, breast cancer cells, diabetes, liver disease, colitis, IBD, radiation-induced intestinal damage, neurotoxicity, ARDS and kidney damage. Clinical studies also showed the antioxidant effect of CM in patients with ASD by increased serum levels of GST and SOD, but decreased MPO.

The immunomodulatory activities of CM and its derivatives in immune disorders of various parts of the body in several experimental studies were shown such as arthritis, liver disease, diabetes, intestinal damage, acute and chronic colitis, neurotoxicity, LPS-induced ARDS and renal disorders. The immunomodulatory properties of CM and its compounds were shown by the effect on various cytokines and immune cells such as Th1 and Th2. Immunomodulatory effects of CM and its components were also demonstrated in clinical studies of chronic hepatitis B and patients with HCV by increasing IFN-γ and Th1 but decreasing IL-4 and Th2, as well as TNF-α level.

**Figure 2:**
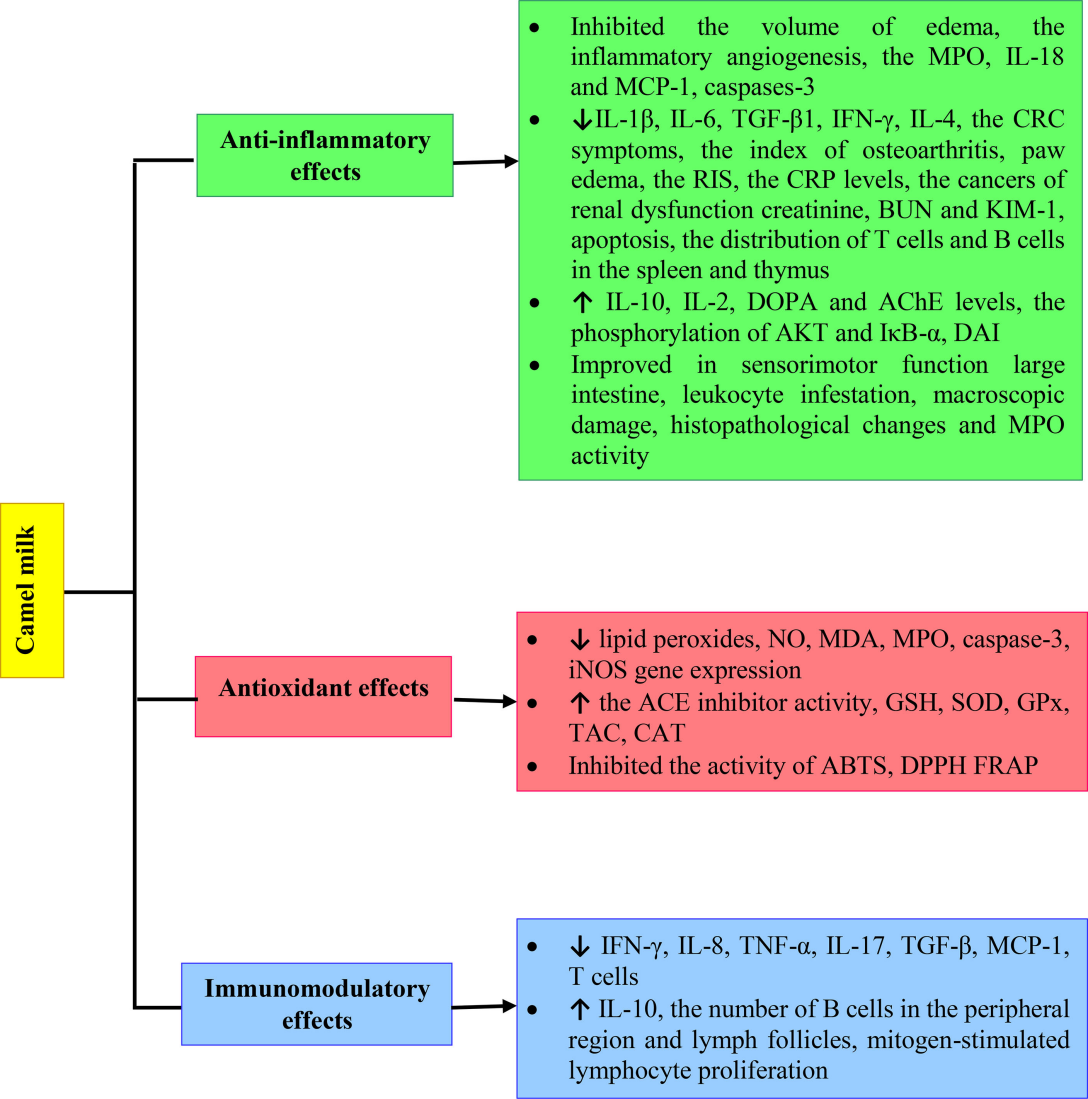
Various mechanisms of anti-inflammatory, antioxidant and immunomodulatory effects of Camel milk.

There is a significant body of research into the health benefits of CM and its derivatives. Here we have discussed the findings collectively and revealed a clear pattern of evidence of CM improving physiological effects of inflammatory diseases, oxidative stress and immune system disorders by increased GSH, SOD, GPx, TAC and CAT levels and reduced TNF-α, IL-17 and TGF-β. However, more extensive clinical studies should be performed on the immunomodulatory, anti-inflammatory and antioxidant effects of CM to better understand the impacts on human health. The various mechanisms of anti-inflammatory, antioxidant and immunomodulatory effects of Camel milk are shown in **Figure 2**.

## Author Contributions

SB, AM, and SS drafted the article and performed the literature research. MB putted forward the idea of the article and critically revised the work. HS and GF reviewed and edited the article. All authors contributed to the article and approved the submitted version.

## Conflict of Interest

The authors declare that the research was conducted in the absence of any commercial or financial relationships that could be construed as a potential conflict of interest.

## Publisher’s Note

All claims expressed in this article are solely those of the authors and do not necessarily represent those of their affiliated organizations, or those of the publisher, the editors and the reviewers. Any product that may be evaluated in this article, or claim that may be made by its manufacturer, is not guaranteed or endorsed by the publisher.

## Glossary

**Table:**

ABTS	3-ethylbenzthiazoline-6-sulphonic acid
ACE	Angiotensin-converting enzyme
AChE	Acetylcholinesterase
ACP	Acid phosphatase
AKT	Protein kinase B
ALP	Alkaline phosphatase
ALT	Alanine aminotransferase
ARDS	Acute respiratory distress syndrome
α-La	Alpha-lactalbumin
ASD	Autism spectrum disorder
AST	Aspartate aminotransferase
β-CN	Beta-Caseins
β-Lg	Beta-lactoglobulin
BUN	Blood urea nitrogen
CAT	catalase
CLA	Conjugated linoleic acid
CM	camel milk
CMPH	Camel milk protein hydrolysis
CRC	Colorectal Cancer
CRP	C reactive protein
CWP	Camel whey protein
CYP	Cyclophosphamide
DAI	Disease activity index
DMWP	Un-denatured whey proteins
DN	Diabetic nephropathy
DNA	Deoxyribonucleic acid
DOPA	Dopamine
DPPH	2;2-diphenyl-1-picryl-hydrazyl-hydrate
DSS	Sodium dextran sulfate
ERK	Extracellular signal-regulated kinases
Fas	Programmed cell death-receptor
FCM	Fermented camel milk
FNP	Fenpropathrin
FRAP	Ferric reducing antioxidant power
GPx	Glutathione peroxidase
GR	Glutathione reductase
GSH	Glutathione
GST	Glutathione transferase
γ-GT	gamma glutamyl transferase
HDL	high-density lipoprotein
HP	Hydroperoxide
IBD	Inflammatory bowel disease
IFN-γ	Interferon gamma
Ig	Immunoglobulin
IκB-α	nuclear factor of kappa light polypeptide gene enhancer in B-cells inhibitor, alpha
IL	Interleukin
iNOS	Inducible nitric oxide synthase
JNK	c-Jun N-terminal kinases
KIM-1	Kidney Injury Molecule-1
LAB	Lactic acid bacteria
LDH	Lactate dehydrogenase
Lf	Lactoferrin
LPS	Lipopolysaccharide
MAPK	Mitogen-activated protein kinases
MCP-1	Monocyte chemoattractant protein-1
MDA	malondialdehyde
MPO	Myeloperoxidase
NAFLD	Nonalcoholic fatty liver disease
NF-κB	Nuclear factor kappa B
NO	Nitric oxide
NOX-1	Nicotinamide adenine dinucleotide phosphate oxidase
PALS	Periarteriolar lymphoid sheaths
PGRP-1	Peptidoglycan recognition protein-1
RA	Rheumatoid arthritis
RIS	Radiation injury score
ROS	Reactive oxygen species
SOD	Superoxide dismutase
TAC	Total antioxidant capacity
T1D	Type 1 diabetes
T2D	Type 2 diabetes
TFA	Total fatty acids
TGF-β1	Transforming growth factor beta 1
Th	T helper
TL	Total lipids
TLR	Toll-like receptor-4
TNBS	Trinitrobenzene sulfonic acid
TNF-α	tumor Necrosis Factor alpha
VEGF	vascular endothelial growth factor
VPA	Valproic acid
